# Predicting Japanese Kampo formulas by analyzing database of medical records: a preliminary observational study

**DOI:** 10.1186/s12911-016-0361-9

**Published:** 2016-09-13

**Authors:** Tetsuhiro Yoshino, Kotoe Katayama, Yuko Horiba, Kaori Munakata, Rui Yamaguchi, Seiya Imoto, Satoru Miyano, Hideki Mima, Kenji Watanabe

**Affiliations:** 1 Center for Kampo Medicine, Keio University School of Medicine, 35 Shinanomachi, Shinjuku-ku, Tokyo, 160-8582 Japan; 2 Human Genome Center, The Institute of Medical Science, The University of Tokyo, 4-6-1 Shirokanedai, Minato-ku, Tokyo, 108-8639 Japan; 3 SFC Laboratory, Keio University, 5322 Endo, Fujisawa, Kanagawa 252-0882 Japan; 4 Division of Health Medical Data Science, Health Intelligence Center, The Institute of Medical Science, The University of Tokyo, 4-6-1 Shirokanedai, Minato-ku, Tokyo, 108-8639 Japan; 5 School of Engineering, The University of Tokyo, 7-3-1 Hongo, Bunkyo-ku, Tokyo, 113-8656 Japan; 6 Faculty of Environment and Information Studies, Keio University, 5322 Endo, Fujisawa, Kanagawa 252-0882 Japan

**Keywords:** Japanese Kampo medicine, Traditional medicine pattern diagnosis, Random forests, Decision support system

## Abstract

**Background:**

Approximately 90 % of physicians in Japan use Kampo medicine in daily practice. However, it is a challenge for physicians who do not specialize in Kampo medicine to select a proper Kampo formula out of the 148 officially approved formulas, as the decision relies on traditional measurements and traditional medicine pattern diagnoses. The present study tries to evaluate the feasibility of a decision support system for frequently used Kampo formulas.

**Methods:**

Our study included 393 patients who visited the Kampo Clinic at Keio University Hospital for the first time between May 2008 and March 2013. We collected medical records through a browser-based questionnaire system and applied random forests to predict commonly prescribed Kampo formulas.

**Results:**

The discriminant rate was the highest (87.0 %) when we tried to predict a Kampo formula from two candidates using age, sex, body mass index, subjective symptoms, and the two essential and predictable traditional medicine pattern diagnoses (excess–deficiency and heat–cold) as predictor variables. The discriminant rate decreased as the candidate Kampo formulas increased, with the greatest drop occurring between three (76.7 %) and four (47.5 %) candidates. Age, body mass index, and traditional medicine pattern diagnoses had higher importance according to the characteristics of each Kampo formula when we utilized the prediction model, which predicted a Kampo formula from among three candidates.

**Conclusions:**

These results suggest that our decision support system for non-specialist physicians works well in selecting appropriate Kampo formulas from among two or three candidates. Additional studies are required to integrate the present statistical analysis in clinical practice.

## Background

The Japanese national health insurance system covers 148 Kampo formulas—which are traditional Japanese herbal formulas mainly derived from ancient China—making them widely available, and each formula has some indications for Western diseases and/or symptoms. Approximately 90 % of Japanese physicians who learned Western medicine use Kampo formulas in daily practice [1, 2]. However, in medical school or continuing medical education, medical students and physicians have limited exposure to Kampo medical education. Thus, it is difficult for most Japanese physicians who do not specialize in Kampo medicine to prescribe Kampo formulas according to the traditional medicine pattern diagnoses and theories of Kampo medicine, which are far different from those of Western medicine. Therefore, the Japanese non-specialist physicians prescribe Kampo formulas according to the indications based on Western diseases and/or symptoms (i.e., *maoto* for influenza, *yokukansan* for dementia, and *shakuyakukanzoto* for muscle cramps [3–7]). This “Western disease based” prescription of Kampo formula is easy to perform for non-specialist physicians but far different from the original way of prescription based on the pattern diagnoses. For example, patients diagnosed with a certain Western disease may have various subjective symptoms and objective findings, and will be classified by pattern diagnoses in Kampo medicine. Moreover, different Kampo formulas can be prescribed for patients with the same pattern diagnosis. Questions about safety, effectiveness, and cost of “Western disease based” prescription of Kampo medicine still remain.

A pattern diagnosis in Kampo medicine refers to the complete clinical presentation of the patient at a given moment in time. Physicians specialized in Kampo medicine use four methods of procedures to make their diagnoses including: inspection, hearing, enquiry, and palpation. Based on the information obtained through these complex procedures, the diagnoses are formed through the process of applying the differential diagnoses for “disharmony symptoms” in the areas of excess–deficiency, heat–cold, and body constituents (Qi, blood, and fluid) to chronic health conditions [8]. However, because there are many ingredients of Kampo formulas and countless traditional medicine pattern diagnoses, it is difficult for non-specialist physicians to accurately and promptly choose a suitable Kampo formula. In this situation, a decision support system (DSS) is required for non-specialist physicians.

We are developing a DSS for non-specialist physicians based on a clinical database created by specialist physicians. This system does not rely on traditional measurement methods such as inspection, hearing, and palpation (including pulse and abdominal examinations), which are difficult for non-specialist physicians to perform. Our DSS comprises two parts: (1) the prediction of the traditional medicine pattern diagnosis, and (2) the prediction of the appropriate Kampo formula. We have already reported on the first part, including the two essential diagnoses—excess–deficiency pattern [9] and heat–cold pattern [10]. However, the second part has not yet been reported on. When the non-specialist physicians can select the appropriate Kampo formula with our DSS, the formula selection will be more safe, efficient, and cost-effective. The standardized and reproducible formula selection will be used for the clinical trials of Kampo medicine which include the idea of pattern diagnoses and proper Kampo formulas.

Herein, we discuss the preliminary results on the use of the DSS for predicting Japanese Kampo formulas for non-specialist physicians.

## Methods

### Patient enrollment

Keio University first introduced a browser-based questionnaire for collecting clinical information in May 2008. The present observational study included patients who made their first visit to the Kampo Clinic at Keio University Hospital between May 2008 and March 2013.

Inclusion criteria were a willingness to be in the study and having more than 20 subjective symptoms as determined by 128 questions in the browser-based questionnaire. We excluded patients who answered “yes” to less than 20 symptoms because they tended to be outliers in our previous research [9]. The participants had to be taking at least one Kampo formula, but not more than two.

Exclusion criteria were missing data on age, sex, body mass index (BMI), subjective symptoms, traditional measurements, information on Western diagnoses, and traditional medicine pattern diagnoses, as well as not being prescribed a Kampo formula. Actually, data collection of BMI was started from January 2012, and most of patients who made their first visit until December 2011 were excluded.

### Data collection

The browser-based questionnaire collected clinical information about patient’s subjective symptoms and symptom severity via visual analogue scales (VAS), along with information on age, sex, BMI, traditional measurements, Western diagnoses based on the International Statistical Classification of Diseases and Related Health Problems (ICD-10), traditional medicine pattern diagnoses, and Kampo formulas prescribed by Kampo specialists. We collected information about patients’ subjective symptoms using a questionnaire comprising 128 binary questions [11]. You can find all the items in Appendix: Tables 4 and 5. Among these questions, 106 were also assessed using a VAS if patients answered with “yes” to the binary portion. To normalize within each patient, we divided each patient’s VAS score by the maximum VAS possible. Data from each question on the traditional measurements, Western diagnoses, traditional medicine pattern diagnoses, and Kampo formulas were binary.

### Model fitting procedure

We applied random forests in accordance with our previous reports [9, 10] to predict the frequently used Kampo formulas. Kampo formula for each data record was based on the browser-based questionnaire, and was selected by a Kampo specialist at the first consultation for the patient in Keio University. The random forests method was developed by Breiman [12, 13] and is a classification algorithm that uses an ensemble of classification trees. Random forests build a large collection of decorrelated trees and then averages them [14]. Random forests often have very good predictive accuracy [15] and have been widely used in many fields (e.g., body pose recognition for Microsoft’s popular Kinect sensor [16]).

We set the number of candidate Kampo formulas from among the most frequent two or more. If there were Kampo formulas with the same frequency, we added either one or all of them as candidates.

We used two sets of predictor variables: the non-specialist variable set and the specialist variable set. In this analysis, we did not perform the variable selection. We intended to validate the difference in discriminant rates between these two variable sets to see if the non-specialist variable set, which did not include all of the items Kampo specialists use in selecting a proper Kampo formula, was appropriate for selecting Kampo formulas. The non-specialist variable set included age, sex, BMI, subjective symptoms, and the two essential and predictable traditional medicine pattern diagnoses—excess–deficiency and heat–cold—according to Kampo specialists. The specialist variable set included ten abdominal examination findings, which are especially important traditional measurement methods, and eight body constituent patterns, in addition to all of the predictor variables of the non-specialist variable set [8, 17].

The two essential patterns of the non-specialist variable set were used because we found that they can be accurately predicted using BMI and subjective symptoms [9, 10]. In contrast, the body constituent patterns were included only in the specialist variable set because they are rather difficult to predict. Abdominal examination findings were also included only in the specialist variable set because it is difficult for non-specialist physicians to perform abdominal examinations without training in Kampo medicine. Furthermore, the findings of abdominal examinations using the traditional method have been reported to vary between observers [18].

An internal validation of the prediction model was based on leave-one-out cross-validation (LOOCV) [19]. In this procedure, we set one patient’s data as validation set and the remaining data as training set. For example about the two candidates, hachimijiogan (54 patients) and kamishoyosan (46 patients), we obtained the prediction model for the Kampo formulas with 99 training data, and predicted Kampo formulas for rest one validation data. We calculated the discriminant rate repeating this procedure for all the 100 data. We did not perform any external validation in this preliminary analysis.

### Variable importance and the marginal effects of random forests

Random forests can calculate the mean decrease in the Gini coefficient of each tree. This is called the *importance.* At each split in each tree, the improvement in the split criterion is an indicator of the importance attributed to the splitting variable; this importance is accumulated over all the trees in the forest separately for each variable. A higher importance means that the variable makes a more sizable contribution to predicting the Kampo formulas.

Random forests also calculate the marginal effect of a variable on the class probability, called the *partial dependency*. A positive value means that the variable contributes positively to selection of a Kampo formula. In contrast, a negative value indicates that the variable contributes negatively to selection of the Kampo formula. Finally, a value of zero indicates that the variable does not contribute at all to the selection of the Kampo formula.

### Statistical analyses

All statistical analyses were conducted with the use of R version 3.1.1 (The R Foundation for Statistical Computing; July 10, 2014, see also: http://www.r-project.org). We used the package “randomForest” [20] and parameters were used as default settings. Data are presented as means ± standard deviations (SDs).

## Results

### Participant information

**Fig. 1:**
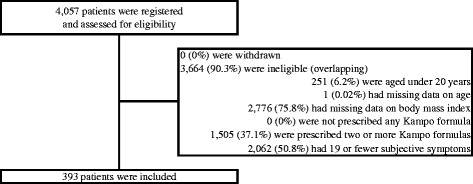
Participant recruitment flow diagram. The largest cause of ineligibility was missing data of on body mass index, which was happened for 2776 patients (75.8 %) who made their first visit between May 2008 and December 2011. Actually, data collection of body mass index was started from January 2012, and most of patients who made their first visit until December 2011 were excluded

We registered 4057 patients who made their first visit to the Kampo Clinic at Keio University Hospital between May 2008 and March 2013. The largest reason of ineligibility was missing data on BMI, which was happened for 2776 patients (75.8 %) who made their first visit until December 2011. About a half of patients had 19 or fewer subjective symptoms, and about one third of patients were prescribed two or more Kampo formulas. Finally, we decided to use data from 393 patients in this analysis, including 57 male and 336 (85.5 %) female (Fig. 1). The mean age was 57.9 ± 16.3 years old, and the mean BMI was 21.5 ± 3.3 kg/m^2^. Of the 393 patients, 17 were categorized as showing an excess pattern, 55 a slight excess pattern, 189 an intermediate (between excess and deficiency) pattern, 79 a slight deficiency pattern, and 53 a deficiency pattern. Furthermore, of the total sample, 16 were categorized as showing a heat pattern, 52 an intermediate (neither heat nor cold) pattern, 214 a cold pattern, and 111 a tangled heat and cold ﻿﻿(both heat and cold)﻿ pattern (Table 1).

**Table 1:** Baseline characteristics of participants

	Eligible patients	Ineligible patients
Number of patients	393	3664
Age at consultation		
Mean ± SD	57.9 ± 16.3	46.2 ± 19.5
Median (Range)	61 (20–91)	44 (0–95)
N/A (%)	0 (0)	1 (0.03)
Male: Female	57:336	1073:2591
BMI, kg/m^2^		
Mean ± SD	21.5 ± 3.3	21.0 ± 3.5
Median (Range)	21.1 (12.8–35.2)	21.0 (12.1–43.9)
N/A (%)	0 (0)	2776 (75.8)
Number of subjective symptoms		
Mean ± SD	32.1 ± 10.2	21.0 ± 12.8
Median (Range)	30 (20–93)	18 (1–99)
N/A (%)	0 (0)	19 (0.5)
Chief complaints (Top 10 in eligible patients)		
Cold sensations	72 (18.3)	455 (12.4)
Insomnia	69 (17.6)	259 (7.1)
Hypertension	62 (15.8)	230 (6.3)
Constipation	42 (10.7)	199 (5.4)
Dyslipidemia	36 (9.2)	61 (1.7)
Depression	29 (7.4)	76 (2.1)
Dizziness	27 (6.9)	69 (1.9)
Chronic gastritis	26 (6.6)	102 (2.8)
Tinnitus	24 (6.1)	76 (2.1)
Headache	22 (5.6)	166 (4.5)
Excess-Deficiency pattern (%)		
Excess pattern	17 (4.3)	276 (7.5)
Slight excess pattern	55 (14.0)	396 (10.8)
Intermediate (between excess and deficiency) pattern	189 (48.1)	1933 (52.8)
Slight deficiency pattern	79 (20.1)	546 (14.9)
Deficiency pattern	53 (13.5)	513 (14.0)
Heat-Cold pattern (%)		
Heat pattern	16 (4.1)	210 (5.7)
Intermediate (neither heat nor cold) pattern	52 (13.2)	1234 (33.7)
Cold pattern	214 (54.5)	1574 (43.0)
Tangled heat and cold ﻿﻿(both heat and cold)﻿ pattern	111 (28.2)	313 (8.5)

Abbreviations: *N/A* not available; *BMI* body mass index, *SD* Standard deviation

*Hachimijiogan* was the most frequently prescribed Kampo formula (for 54 patients) in our data set, followed by *kamishoyosan* (46 patients), *keishikaryukotsuboreito* (33 patients), and *keishibukuryogan* (29 patients). *Shimbuto*, *bukuryoingohangekobokuto*, and *yokukansan* were each used for 17 patients. We summarized the top 10 frequently used Kampo formulas in Table 2. The mean age for patients prescribed hachimijiogan was the highest of the ten Kampo formulas, and the mean BMI for patients prescribed keishikaryukotsuboreito was the lowest of the ten formulas.

**Table 2:** Demographics and standard traditional pattern diagnosis of frequently used Kampo formulas

	Number	Mean age ± SD	Male: Female	Mean BMI ± SD	Excess-Deficiency	Heat-Cold	Body constituents (Qi-Blood-Fluid)
Hachimijiogan	54	71.9 ± 9.6	19:35	22.9 ± 3.1	Deficiency	Cold	Fluid disturbance
Kamishoyosan	46	53.5 ± 13.3	0:46	22.2 ± 2.4	Deficiency	Tangled heat and cold	Qi counterflow Blood stasis
Keishikaryukotsuboreito	33	56.3 ± 14.1	2:31	18.6 ± 2.2	Deficiency	Intermediate	Qi counterflow
Keishibukuryogan	29	49.0 ± 15.1	0:29	23.2 ± 3.4	Intermediate	Intermediate	Qi counterflow Blood stasis
Shimbuto	17	62.4 ± 18.4	2:15	20.2 ± 4.0	Deficiency	Cold	Fluid disturbance
Bukuryoingohangekobokuto	17	55.6 ± 14.3	1:16	20.7 ± 3.6	Deficiency	Intermediate	Qi stagnation Fluid disturbance
Yokukansan	17	51.9 ± 16.0	2:15	21.3 ± 2.6	Intermediate	Heat	Not defined
Tokishakuyakusan	15	49.1 ± 16.4	0:15	20.2 ± 1.5	Deficiency	Cold	Blood stasis Fluid disturbance
Hochuekkito	12	60.6 ± 16.1	2:10	22.0 ± 2.7	Deficiency	Intermediate	Qi deficiency
Tokishigyakukagoshuyushokyoto	11	58.1 ± 13.7	0:11	22.6 ± 3.0	Deficiency	Cold	Not defined
Overall	393	57.9 ± 16.3	56:337	21.5 ± 3.3			

Abbreviations: *BMI* body mass index, *SD* Standard deviation

### Model fitting procedure

**Fig. 2:**
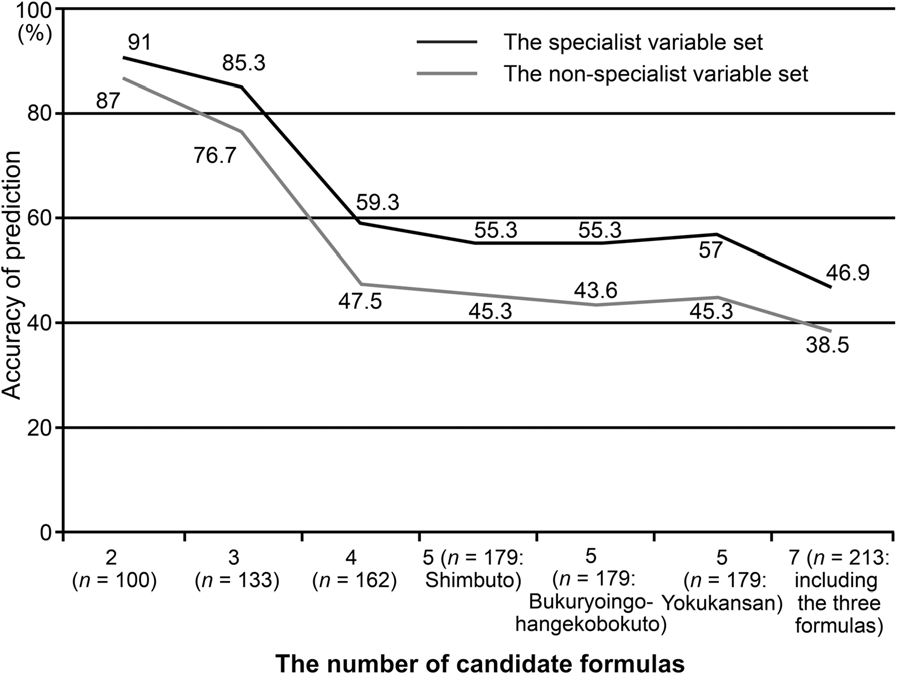
Leave-one-out cross-validation accuracy of prediction according to the number of candidate Kampo formulas. The discriminant rate via leave-one-out cross-validation was highest when we tried to predict a Kampo formula from among two candidates, hachimijiogan and kamishoyosan. The discriminant rate decreased as the number of candidate Kampo formulas increased; in particular, it showed a marked drop in shifting from three to four candidates, meaning when we added the fourth Kampo formula keishibukuryogan to the top three formulas, hachimijiogan, kamishoyosan, and keishikaryukotsuboreito (see also Table 2). The non-specialist variable set included age, sex, body mass index, and subjective symptoms, as well as the two essential and predictable traditional medicine pattern diagnoses (excess–deficiency and heat–cold) according to Kampo specialists. The specialist variable set included abdominal examination findings and body constituent patterns in addition to all of the predictor variables of the non-specialist variable set

We applied random forests for the prediction of Kampo formulas from the selected candidates using either the non-specialist or specialist variable set. The discriminant rate via LOOCV was highest when we tried to predict a Kampo formula from among two candidates, hachimijiogan and kamishoyosan (Fig. 2). The discriminant rate decreased as the number of candidate Kampo formulas increased; in particular, it showed a marked drop in shifting from three to four candidates, meaning when we added the fourth Kampo formula keishibukuryogan to the top three formulas, hachimijiogan, kamishoyosan, and keishikaryukotsuboreito (see also Table 2).

The discriminant rates were 4 to 11.7 % higher when we used the specialist variable set than when using the non-specialist variable set. This gap was smallest when we tried to predict Kampo formulas from among two candidates.

### Variable importance and marginal effects from random forests

Non-specialists would appear to benefit from having fewer candidates when selecting Kampo formulas. In addition, the discriminant rate dropped considerably when the number of candidate Kampo formulas reached four as noted in the previous section. As such, for the non-specialist variable set, we selected a prediction model wherein Kampo formulas were predicted from among three candidates. We then analyzed how this model worked with our data set.

**Fig. 3:**
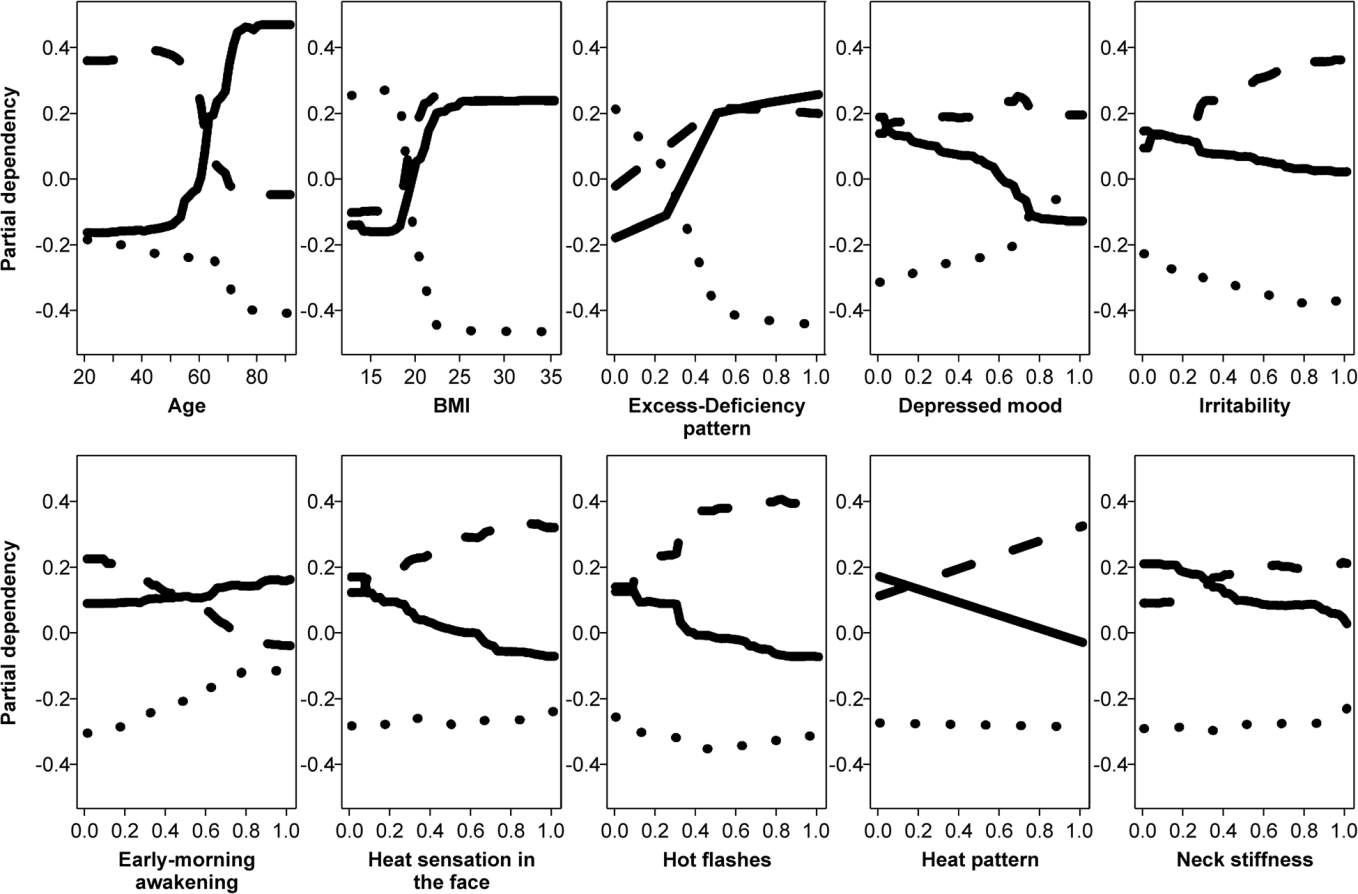
Partial dependency of the top 10 important variables for each Kampo formula. This figure shows the partial dependency plot about the top 10 important variables in Table 3. A positive value in the plot means that the variable contributes positively to selection of the Kampo formula. In contrast, a negative value indicates that the variable contributes negatively to selection of the Kampo formula. A value of zero indicates the variable does not contribute at all to the Kampo formula. Older age, higher body mass index, excess pattern, lower depressed mood and irritability, higher early-morning awakening, lower heat sensation in the face and hot flashes, negative heat pattern, and lower neck stiffness indicated the use of hachimijiogan. In the same way, younger age, higher body mass index, excess pattern, depressed mood, higher irritability, lower early-morning awakening, higher heat sensation in the face and hot flashes, heat pattern, and higher neck stiffness indicated the use of kamishoyosan. Lower body mass index and deficiency pattern indicated keishikaryukotsuboreito, but all other items in the top 10 negatively contributed to use of this Kampo formula. Solid lines are used for hachimijiogan, broken lines for kamishoyosan, and dotted lines for keishikaryukotsuboreito

Among the top 30 items in terms of *importance*, we found that age and BMI had the highest and second highest values, respectively (Table 3). Here, we focused on the top 10 variables with higher importance, and analyzed how they worked in this model. Figure 3 shows the partial dependency plot about the top 10 important variables in Table 3. For example, in the upper left plot for age and BMI, the solid line indicates that those with an age of greater than 60 and a BMI over 20 had a positive value for hachimijiogan. In other words, the model suggests that hachimijiogan be selected when the patients are older than 60 and have a BMI over 20. In contrast, the model suggests that hachimijiogan is not as suitable when the patients are younger than 60 and have a BMI under 20. Similarly, an age under 60 and a BMI over 20 suggests that kamishoyosan be selected, while a BMI under 20 indicates the selection of keishikaryukotsuboreito. The partial dependency of age for keishikaryukotsuboreito was consistently negative, meaning that all ages contributed negatively to selection of this Kampo formula, especially ages over 60 (Fig. 3; age and BMI). These findings indicate that an age of around 60 and a BMI of around 20 did not contribute to the selection of Kampo formulas, except for negatively affecting selection of keishikaryukotsuboreito. These findings on the partial dependency of age and BMI were consistent with the characteristics of patients prescribed the three Kampo formulas shown in Table 2.

**Table 3:** Top 30 most important variables for predicting top 3 Kampo formulas with non-specialist variable set. Higher importance values indicate that the item makes a more sizable contribution to predicting the Kampo formulas

No.	Item name	Importance
1	Age	6.96
2	Body mass index	5.78
3	Excess–deficiency pattern	4.92
4	Depressed mood	2.17
5	Irritability	2.07
6	Early-morning awakening	2.00
7	Heat sensation in the face	1.98
8	Hot flashes	1.96
9	Heat pattern	1.95
10	Neck stiffness	1.73
11	Sex	1.57
12	Bleary eyes	1.57
13	Palpitations	1.50
14	Tires easily	1.49
15	Eyestrain	1.40
16	Leg fluctuation	1.29
17	Arousal during sleep	1.22
18	Headache	1.21
19	Difficulty in falling asleep	1.21
20	Shoulder stiffness	1.20
21	Leg spasms	1.08
22	Forgetfulness	1.06
23	Shoulder pain	1.00
24	Hie legs	0.89
25	Quick to sweat	0.89
26	Back stiffness	0.87
27	Decreased visual acuity	0.86
28	Sleepy after eating	0.85
29	Numbness legs	0.82
30	Dry skin	0.81

**Fig. 4:**
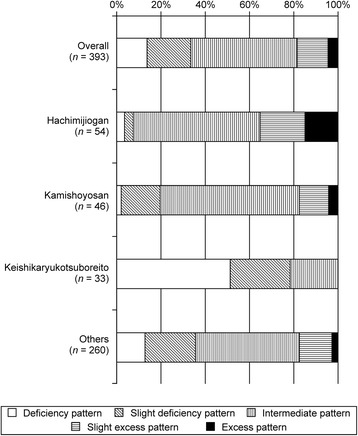
Excess–deficiency pattern diagnoses of patients by Kampo specialists. Kampo specialists rarely prescribed hachimijiogan for treating patients diagnosed with a deficiency pattern or a slight deficiency pattern. In contrast, keishikaryukotsuboreito was not prescribed for patients diagnosed with a slight excess pattern or an excess pattern

**Fig. 5:**
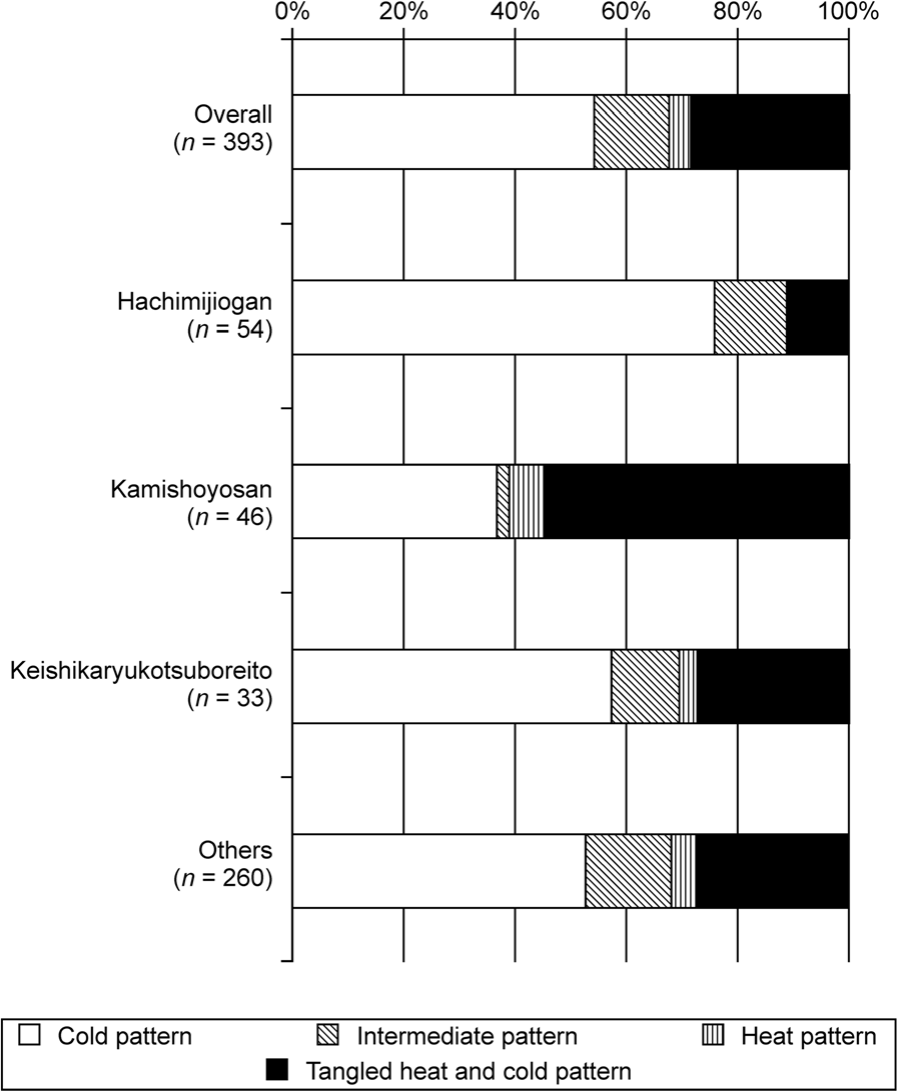
Heat–cold pattern diagnoses of patients by Kampo specialists. Patients who were diagnosed with a heat pattern or a tangled heat and cold pattern by Kampo specialists were rare among patients who were prescribed hachimijiogan. In contrast, over 60 % of patients who were prescribed kamishoyosan were diagnosed with a heat pattern or a tangled heat and cold pattern

We also found that the two traditional medicine pattern diagnoses, excess–deficiency and heat–cold, had high importance (Table 3). The partial dependency plot for excess–deficiency patterns showed that the deficiency pattern indicated use of keishikaryukotsuboreito and not hachimijiogan. In contrast, the excess pattern suggested the use of hachimijiogan or kamishoyosan and not keishikaryukotsuboreito (Fig. 3; excess–deficiency pattern). In fact, Kampo specialists rarely prescribed hachimijiogan for treating patients diagnosed with a deficiency or a slight deficiency pattern. In contrast, keishikaryukotsuboreito was not prescribed for patients diagnosed with a slight excess pattern or an excess pattern (Fig. 4). The partial dependency plot for the heat pattern showed that the heat pattern indicated the use of kamishoyosan, and not hachimijiogan or keishikaryukotsuboreito (Fig. 3; heat pattern). Patients who were diagnosed with a heat pattern or a tangled heat and cold pattern by Kampo specialists were generally not prescribed hachimijiogan. In contrast, over 60 % of patients who were prescribed kamishoyosan were diagnosed as having a heat pattern or a tangled heat and cold pattern (Fig. 5).

**Fig. 6:**
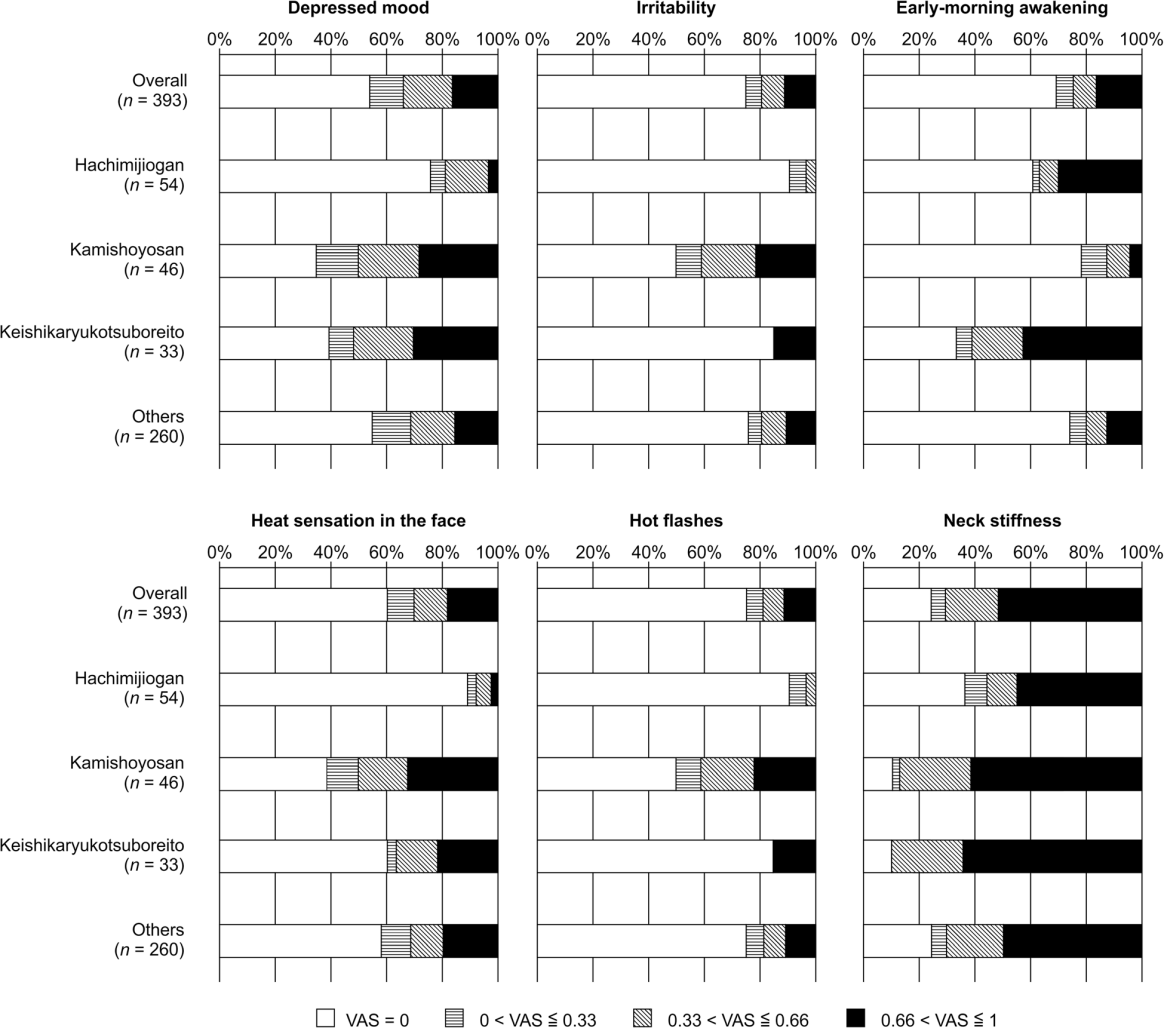
Severity of subjective symptoms with top 10 importance rankings. Many of the patients who were prescribed hachimijiogan did not have depressed mood, irritability, heat sensation in the face, or hot flashes. They also tended to not have neck stiffness. In contrast, many patients who were prescribed kamishoyosan had a more depressed mood, irritability, heat sensation in the face, and hot flashes, but did not exhibit early-morning awakening. Patients who were prescribed keishikaryukotsuboreito tended to have a more depressed mood and early-morning awakening and to not have irritability or hot flashes

In a similar manner with the subjective symptoms, lack of depressed mood and irritability; early-morning awakening; and lack of heat sensation in the face, hot flashes, or neck stiffness indicated the use of hachimijiogan. In contrast, presence of depressed mood and irritability; lack of early-morning awakening; and presence of hot flashes, heat sensation in the face, and neck stiffness indicated the use of kamishoyosan. Lack of irritability and hot flashes and the presence of a depressed mood and early-morning awakening indicated the use of keishikaryukotsuboreito (Fig. 3; depressed mood, irritability, early-morning awakening, heat sensation in the face, hot flashes, and neck stiffness). This was consistent with the results related to the severity of symptoms summarized in Fig. 6. Many of the patients who were prescribed hachimijiogan did not have a depressed mood, irritability, heat sensation in the face, or hot flashes. They also tended not to have neck stiffness. In contrast, many patients who were prescribed kamishoyosan exhibited a depressed mood, irritability, heat sensation in the face, and hot flashes, but did not exhibit early-morning awakening. Patients who were prescribed keishikaryukotsuboreito tended to exhibit a more depressed mood and early-morning awakening and to not exhibit irritability or hot flashes.

To further understanding, we provided the results from multinomial logistic regression when we tried to predict a Kampo formula from among the same three candidates with using non-specialist variable set in Appendix: Tables 4 and 5. You can obtain area under the receiver operating characteristic curves﻿, and﻿ crude odds ratios with 95 % confidential intervals for each item of non-specialist variable set.

## Discussion

Approximately 90 % of Japanese physicians who are well educated in Western medicine use Kampo formulas regularly despite not having specific education in Kampo medicine. We believe that they will benefit most from the DSS to make more standardized traditional medicine pattern diagnoses and prescribe appropriate Kampo formulas [2]. In this article, we discussed the preliminary results of our DSS in predicting use of appropriate Kampo formulas.

In this study, we used only 9.7 % of overall patients’ data. Although it is uncommon, we provided the baseline characteristics of participants not only about eligible patients but also ineligible patients. Our data were primarily obtained from female patients, and this sex difference is common in Japanese Kampo clinics [21, 22]. This is because some Kampo formulas, including kamishoyosan and keishibukuryogan, are especially used for female patients. These Kampo formulas are originally designed for perimenopausal or post-partum women, respectively. We could obtain similar results using only data from female patients. When we excluded male patients, female sex was disappeared from the table of important variables but the other variables had almost the same rank with same value of *importance* (Data not shown).

Our results suggested that a greater number of candidate Kampo formulas led to a worse discriminant rate. Specifically, the DSS could only handle around two or three frequently used Kampo formulas, even though there are far more officially approved formulas in Kampo medicine. Indeed, upon including a fourth Kampo formula, keishibukuryogan, the discriminant rate dropped by approximately 30 %. This was likely because it was difficult to differentiate between the second Kampo formula, kamishoyosan, and the fourth, keishibukuryogan (data not shown). Our data suggest that differences between the top three Kampo formulas were rather large, whereas the differences between these top three formulas and the more minor formulas were relatively small. If our DSS could handle only these two or three candidate Kampo formulas, it would not be able to support non-specialist physicians in daily practice.

Our DSS might be able to cover more Kampo formulas using cluster analysis, which has already given us other candidate formulas [23]. Our DSS will support non-specialist physicians in daily practice when the DSS will be combined with the cluster analysis. We have already reported that cluster analysis can reproduce some traditional medicine pattern diagnoses, and that frequently used Kampo formulas differed among each cluster, in accordance with the traditional medicine pattern diagnoses they best suited [23]. In other words, our model can be applied to different clusters of diagnoses, which may help us obtain the most appropriate Kampo formulas from among those most frequently used. However, there are only a few papers reporting the results of a cluster analysis on this topic. Ishizuka et al. also reported on the results of a cluster analysis of patients who visited a Kampo institution in Japan. They concluded that their cluster analysis results supported the rationale behind the empirical determination of traditional medicine pattern diagnosis [24]. However, their results supported only kidney and liver deficiency patterns, and did not discuss Kampo formulas. There are two more articles reporting cluster analyses, but they are not widely accessible because they are written in Chinese [25, 26].

We utilized two sets of predictor variables, and found that the non-specialist variable set worked well in predicting appropriate Kampo formulas. Indeed, the discriminant rates were more affected by the number of candidate Kampo formulas than by the change in the variable set. We found that we could achieve higher discriminant rates when we used the specialist variable set including the abdominal examination findings and the body constituent patterns. However, the findings from traditional measurements can vary between physicians, and thus the standardization of such traditional measurements remains an issue [27]. Use of standardized traditional measurements will help also in the prediction of body constituent patterns.

We analyzed the importance and partial dependency of the variables. It must be noted that the variables with high importance are useful only for the random forests and would not directly relate to the clinical decisions of Kampo specialists. However, we noted that many of these items were compatible with our clinical experience. While this experience is based on ancient knowledge and traditional theory, a significant amount of it has proven relevant today. For example, Odaguchi et al., in 2007, reported that a Kampo formula, goshuyuto, was effective for some patients with headaches with specific characteristics; these characteristics were similar to those written about in traditional medical textbooks [28].

We also tried using logistic regression with Lasso penalty as a linear model [29] and classification and regression trees (CART) as a non-linear model [30], but random forests performed the best in terms of the discriminant rate. For example, the discriminant rates were 85.0 and 79.0 % by logistic regression, or by CART, respectively, when we tried to predict a Kampo formula from among two candidates with non-specialist variable set. In contrast, the discriminant rates via LOOCV were both 90.0 % with specialist variable set by logistic regression methods, or by CART. From this result, the statistical methods are not important issue with enough predictors, but are important with restricted predictors.

Herein, we predicted specialists’ selections of Kampo formulas in daily clinical situations, and the selection didn’t depend on an objective golden standard or pre-defined standards. The Kampo specialists select a “proper” Kampo formula based on traditional medicine pattern diagnosis and theory. We provided the standard pattern diagnosis for each Kampo formula defined by the Japanese Society of Oriental Medicine in Table 2. However, the diagnosis in the table was not the only one way, and different pattern diagnosis can be made for the patients with the same Kampo formulas as shown in the Figs. 4 and 5. We can understand the actual connection between Kampo formula and traditional medicine pattern diagnosis with this study, but we can also recognize a problem about standardization of diagnosis and Kampo formula selection. One possible reason of this discrepancy is the indications for each Kampo formula do not include traditional medicine pattern diagnosis; rather they rely only on Western diseases and symptoms.

Additionally, we did not consider efficacy. “True” Kampo formulas must be defined by their efficacy. Although most prescriptions by specialists are effective, sometimes there may be better options. We have already noticed that a few patients got better after changing the Kampo formula which was selected at the first consultation. Then, we must carefully confirm the proper Kampo formula, especially for patients whose formulas were difficult to predict.

We did not perform variable selection in this analysis, and used many variables for small number of patients. The prediction models with small number of variables may be better for generalization like applying this method to other medical record databases. We, however, showed the results with all of the potential variables because we thought it would help non-specialist physicians to understand specialists’ tacit knowledge (See also Appendix: Tables 4 and 5).

Our aim is to further progress this DSS reflecting clinical outcomes and the experience of other institutions. Now, we are collecting clinical information through the browser-based questionnaire at some representative institutions about Kampo medicine. We have collected more than forty thousand of record from more than eight thousand patients. This data will further progress our DSS.

## Conclusions

Our decision support system for non-specialist physicians works well in selecting a proper Kampo formula from two or three candidates. Additional studies are required to integrate such statistical analysis in clinical practice.

## Appendix

**Table 4:** Area under the receiver operating characteristic curve for each combination of formulas. Area under the curve (AUC) was calculated using the R package “pROC”. A value of 1 for the dependent variable (case) denotes the Kampo formula listed in the upper row. A value of 0 for the dependent variable (control) denotes the Kampo formula listed in the bottom row. AUC > 0.5 indicates preferential use of the Case Kampo formula when the patient gave a positive response (or relatively higher response in the case of visual analogue scales) for each item. In contrast, AUC < 0.5 indicates use of the Control Kampo formula when the patient gave a positive response. Items with AUC values over 0.7 or under 0.3 are shown in bold, indicating strongly suggestive results. Item names with (Bi) means binary questions, and without (Bi) means questions with visual analogue scales

Item name	Area under the curve		
1 (case) 0 (control)	Hachimijiogan vs Kamishoyosan	Kamishoyosan vs Keishikaryukotsuboreito	Hachimijiogan vs Keishikaryukotsuboreito
Subjective symptoms			
Appetite			
Appetite loss (Bi)	0.506	0.355	0.361
Good appetite (Bi)	0.521	0.513	0.535
Speed of the meal			
Slow speed of the meal (Bi)	0.515	0.496	0.511
Fast speed of the meal (Bi)	0.502	0.518	0.520
Difficulty falling asleep	0.610	0.319	0.442
Arousal during sleep	0.562	0.307	0.372
Early-morning awakening	0.611	**0.242**	0.385
I dream frequently (Bi)	0.424	0.429	0.353
Single dose of urine large (Bi)	0.497	0.507	0.503
Single dose of urine low (Bi)	0.555	0.474	0.529
Difficulty urinating	0.554	0.511	0.565
Urination pain	0.509	0.500	0.509
Urine leakage	0.484	0.573	0.552
Enuresis	0.500	0.500	0.500
Hard stool (Bi)	0.490	0.535	0.525
Small and round stool (Bi)	0.400	0.568	0.468
Soft stool (Bi)	0.442	0.475	0.417
Diarrhea	0.490	0.456	0.446
Hard to stool (Bi)	0.546	0.489	0.535
Hemorrhoid	0.486	0.484	0.474
Anal prolapse	0.528	0.485	0.513
Bloody stool	0.489	0.496	0.485
Taking laxatives (Bi)	0.527	0.505	0.532
Depressed mood	**0.267**	0.491	**0.291**
Forgetfullness	0.488	0.403	0.393
Irritated	**0.287**	0.698	0.496
Dry skin	0.391	0.527	0.428
Itchy skin	0.508	0.544	0.564
Acne	0.457	0.513	0.470
Blot	0.378	0.524	0.405
Urticaria	0.442	0.561	0.505
Wart	0.484	0.498	0.482
Athlete’s foot	0.542	0.522	0.566
Brittle nails	0.415	0.541	0.458
Get tired easily	0.359	0.439	**0.296**
Easy to sweat	0.364	0.573	0.449
Night sweats	0.477	0.503	0.479
Hot flush	**0.286**	0.648	0.464
Heat intolerance	0.426	0.580	0.522
Cold intolerance	0.497	0.427	0.428
Attenuation of sexual desire	0.505	0.447	0.449
Impotence (male)	0.519	0.485	0.504
Neck stiffness	0.353	0.484	0.356
Shoulder stiffness	0.402	0.438	0.362
Back stiffness	0.464	0.418	0.401
Lower back stiffness	0.469	0.470	0.440
Facial pain	0.500	0.485	0.485
Hand pain	0.504	0.459	0.462
Foot pain	0.605	0.455	0.562
Shoulder pain	0.452	0.445	0.405
Back pain	0.481	0.501	0.482
Hip pain	0.593	0.498	0.586
Knee pain	0.595	0.564	0.648
Numbness face	0.478	0.477	0.455
Numbness hands	0.538	0.489	0.531
Numbness legs	0.615	0.501	0.617
Numbness back	0.498	0.481	0.478
Trembling face	0.489	0.511	0.500
Trembling hands	0.513	0.447	0.461
Trembling legs	0.478	0.507	0.485
Hie general	0.452	0.529	0.482
Hie hands	0.465	0.439	0.403
Hie legs	0.550	0.459	0.491
Hie lower back	0.520	0.460	0.482
Heat face	**0.240**	0.595	0.348
Heat hands	0.446	0.539	0.485
Heat legs	0.455	0.540	0.494
Edema face	0.454	0.565	0.513
Edema hands	0.464	0.570	0.531
Edema legs	0.458	0.534	0.495
Headache	0.357	0.604	0.457
Sluggishness	0.405	0.482	0.400
Vertigo	0.480	0.495	0.477
Lightheadedness	0.432	0.514	0.452
Dandruff	0.544	0.466	0.510
Hair loss	0.430	0.522	0.449
Decreased visual acuity	0.457	0.492	0.455
Eyestrain	0.338	0.549	0.401
Blurred vision	0.442	0.559	0.500
Bleary eyes	**0.298**	0.604	0.411
Dark circles of the eyes	0.407	0.512	0.425
Sneezing	0.497	0.560	0.555
White nasal discharge (Bi)	0.467	0.477	0.444
Yellow nasal discharge (Bi)	0.508	0.511	0.519
Post nasal drip	0.496	0.426	0.418
Stuffy nose	0.533	0.445	0.477
Nosebleed	0.489	0.481	0.470
Mouth bitter	0.490	0.468	0.457
Saliva comes out	0.488	0.522	0.509
Throat pain	0.482	0.482	0.464
Throat jams	0.395	0.583	0.481
Thirsty	0.529	0.493	0.523
Dry mouth	0.456	0.461	0.420
Dry lips	0.496	0.423	0.413
Take water often	0.468	0.522	0.496
Tinnitus	0.491	0.536	0.527
Hearing loss	0.637	0.481	0.618
Cough	0.477	0.509	0.485
White sputum (Bi)	0.562	0.431	0.492
Yellow sputum (Bi)	0.517	0.481	0.497
Asthma	0.476	0.533	0.509
Shortness of breath	0.512	0.417	0.428
Palpitation	0.368	0.414	**0.280**
Chest pain	0.503	0.466	0.470
Burp	0.429	0.525	0.457
Heartburn	0.508	0.522	0.533
Epigastric jamming discomfort	0.494	0.451	0.445
Nausea	0.477	0.469	0.448
Vomiting	0.500	0.485	0.485
Motion sickness	0.489	0.508	0.500
Stomach fullness	0.498	0.518	0.513
Stomach rumbling	0.476	0.408	0.386
Flaturence	0.537	0.507	0.547
Sleepy after eating	0.393	0.587	0.481
Abdominal pain	0.525	0.409	0.438
Abdominal pain fasting (Bi)	0.519	0.455	0.473
Abdominal pain after eating (Bi)	0.509	0.455	0.464
Abdominal pain at upper (Bi)	0.500	0.470	0.470
Abdominal pain at lower (Bi)	0.526	0.465	0.492
Hand stiffness	0.460	0.556	0.513
Lower extremities weakness	0.589	0.450	0.543
Legs fluctuates	0.669	0.466	0.637
Leg spasms	0.598	0.541	0.655
Frost bite	0.487	0.517	0.504
Heavy menstrual flow (Female, Bi)	0.435	0.565	0.500
Less menstrual flow (Female, Bi)	0.435	0.489	0.424
Menstruation textile (Female)	0.370	0.533	0.409
Menstrual pain (Female)	0.359	0.586	0.439
Irregular menstruation (Female, Bi)	0.402	0.507	0.409
Delivery (Female, Bi)	0.466	0.460	0.426
Spontaneous abortion (Female, Bi)	0.458	0.537	0.495
Induced abortion (Female, Bi)	0.514	0.472	0.486
Pregnancy toxemia (Female, Bi)	0.498	0.496	0.494
Abnormal bleeding (Female, Bi)	0.478	0.507	0.485
Traditional pattern diagnosis			
Excess-Deficiency pattern (Five categories)	0.624	**0.856**	**0.899**
Cold pattern (Bi)	0.479	0.532	0.511
Heat pattern (Bi)	**0.251**	0.653	0.404
Background			
Female sex (Bi)	0.324	0.530	0.354
Age	**0.861**	0.422	**0.839**
Body mass index	0.589	**0.878**	**0.887**

**Table 5:** Crude odds ratios for each combination of Kampo formulas. To measure the effect size of each predictive variable, we performed multinomial logistic regression. Odds ratio (OR) was calculated using the R package “nnet”. A value of 1 for the dependent variable (case) denotes the Kampo formula listed in the upper row. A value of 0 for the dependent variable (control) denotes the Kampo formula listed in the bottom row. Item names with (Bi) means binary questions, and without (Bi) means questions with visual analogue scales

Item name	Crude odds ratio (95 % confidence intervals)		
1 (case) 0 (control)	Hachimijiogan vs Kamishoyosan	Kamishoyosan vs Keishikaryukotsuboreito	Hachimijiogan vs Keishikaryukotsuboreito
Subjective symptoms			
Appetite			
Appetite loss (Bi)	1.294 (0.207, 8.099)	0.091 (0.019, 0.446)	0.118 (0.030, 0.463)
Good appetite (Bi)	1.564 (0.427, 5.722)	1.476 (0.254, 8.578)	2.309 (0.450, 11.848)
Speed of the meal			
Slow speed of the meal (Bi)	1.215 (0.443, 3.336)	0.947 (0.295, 3.046)	1.151 (0.381, 3.476)
Fast speed of the meal (Bi)	1.028 (0.398, 2.659)	1.250 (0.404, 3.864)	1.286 (0.431, 3.835)
Difficulty falling asleep	3.755 (1.231, 11.453)	0.175 (0.052, 0.591)	0.657 (0.234, 1.844)
Arousal during sleep	1.912 (0.663, 5.517)	0.158 (0.049, 0.510)	0.302 (0.104, 0.882)
Early-morning awakening	6.605 (1.708, 25.547)	0.051 (0.012, 0.218)	0.340 (0.117, 0.991)
I dream frequently (Bi)	0.378 (0.136, 1.050)	0.535 (0.208, 1.372)	0.202 (0.071, 0.579)
Single dose of urine large (Bi)	0.846 (0.114, 6.256)	1.455 (0.126, 16.743)	1.231 (0.107, 14.128)
Single dose of urine low (Bi)	2.114 (0.732, 6.107)	0.675 (0.197, 2.315)	1.427 (0.483, 4.212)
Difficulty urinating	14.328 (0.269, 762.157)	62249680000.000 (0.000, Inf)	1029688000000.000 (0.000, Inf)
Urination pain	13075.100 (0.000, Inf)	0.049 (0.000, Inf)	428.912 (0.000, Inf)
Urine leakage	2.761 (0.258, 29.497)	8.399 (0.143, 492.448)	23.184 (0.463, 1161.637)
Enuresis	1.000 (1.000, 1.000)	1.000 (1.000, 1.000)	1.000 (1.000, 1.000)
Hard stool (Bi)	0.833 (0.249, 2.786)	2.325 (0.439, 12.321)	1.938 (0.367, 10.219)
Small and round stool (Bi)	0.326 (0.124, 0.856)	1.981 (0.706, 5.559)	0.646 (0.210, 1.985)
Soft stool (Bi)	0.508 (0.194, 1.328)	0.788 (0.299, 2.073)	0.400 (0.145, 1.107)
Diarrhea	0.664 (0.055, 8.056)	0.308 (0.032, 2.974)	0.204 (0.020, 2.116)
Hard to stool (Bi)	1.905 (0.652, 5.561)	0.840 (0.233, 3.025)	1.600 (0.508, 5.041)
Hemorrhoid	2.321 (0.191, 28.221)	0.313 (0.022, 4.417)	0.726 (0.078, 6.721)
Anal prolapse	63127200000.000 (0.000, Inf)	0.000 (0.000, Inf)	10.162 (0.019, 5537.952)
Bloody stool	0.000 (0.000, Inf)	1.265 (0.016, 97.879)	0.000 (0.000, Inf)
Taking laxatives (Bi)	1.515 (0.505, 4.547)	1.087 (0.281, 4.205)	1.648 (0.472, 5.755)
Depressed mood	0.048 (0.010, 0.225)	0.836 (0.215, 3.248)	0.040 (0.008, 0.208)
Forgetfullness	0.816 (0.184, 3.615)	0.237 (0.049, 1.132)	0.193 (0.042, 0.895)
Irritated	0.076 (0.018, 0.318)	10.981 (2.154, 55.976)	0.834 (0.151, 4.604)
Dry skin	0.330 (0.103, 1.062)	1.259 (0.364, 4.346)	0.416 (0.115, 1.498)
Itchy skin	0.951 (0.294, 3.082)	1.612 (0.401, 6.488)	1.534 (0.396, 5.946)
Acne	0.000 (0.000, Inf)	1.581 (0.036, 68.993)	0.000 (0.000, Inf)
Blot	0.276 (0.080, 0.958)	1.240 (0.358, 4.300)	0.343 (0.088, 1.333)
Urticaria	0.154 (0.020, 1.157)	3.170 (0.415, 24.193)	0.488 (0.043, 5.517)
Wart	0.508 (0.052, 4.934)	0.960 (0.102, 9.032)	0.488 (0.043, 5.562)
Athlete’s foot	1.611 (0.171, 15.202)	1.042 (0.067, 16.120)	1.678 (0.135, 20.941)
Brittle nails	0.312 (0.089, 1.103)	1.561 (0.417, 5.838)	0.488 (0.119, 2.004)
Get tired easily	0.250 (0.084, 0.738)	0.519 (0.135, 1.994)	0.129 (0.035, 0.475)
Easy to sweat	0.256 (0.086, 0.763)	1.847 (0.583, 5.856)	0.472 (0.140, 1.598)
Night sweats	0.367 (0.053, 2.539)	1.416 (0.194, 10.350)	0.520 (0.059, 4.598)
Hot flush	0.022 (0.003, 0.182)	4.207 (1.003, 17.643)	0.091 (0.009, 0.896)
Heat intolerance	0.348 (0.110, 1.106)	2.768 (0.729, 10.504)	0.964 (0.243, 3.825)
Cold intolerance	0.951 (0.364, 2.483)	0.527 (0.180, 1.545)	0.501 (0.177, 1.423)
Attenuation of sexual desire	0.807 (0.078, 8.353)	0.222 (0.030, 1.624)	0.179 (0.024, 1.327)
Impotence (male)	10052440000.000 (0.000, Inf)	0.000 (0.000, Inf)	8.044 (0.030, 2144.688)
Neck stiffness	0.220 (0.074, 0.652)	1.142 (0.321, 4.066)	0.251 (0.077, 0.823)
Shoulder stiffness	0.356 (0.119, 1.062)	0.671 (0.175, 2.563)	0.239 (0.067, 0.852)
Back stiffness	0.695 (0.250, 1.932)	0.539 (0.177, 1.645)	0.375 (0.125, 1.127)
Lower back stiffness	0.798 (0.290, 2.195)	0.743 (0.244, 2.258)	0.592 (0.199, 1.763)
Facial pain	0.077 (0.000, Inf)	0.001 (0.000, Inf)	0.001 (0.000, Inf)
Hand pain	1.879 (0.276, 12.784)	0.201 (0.030, 1.366)	0.378 (0.074, 1.932)
Foot pain	6.765 (1.390, 32.926)	0.374 (0.060, 2.339)	2.529 (0.615, 10.389)
Shoulder pain	0.360 (0.086, 1.504)	0.497 (0.142, 1.746)	0.179 (0.043, 0.754)
Back pain	0.590 (0.102, 3.413)	0.734 (0.132, 4.071)	0.433 (0.071, 2.641)
Hip pain	2.550 (0.930, 6.989)	0.863 (0.264, 2.825)	2.200 (0.732, 6.607)
Knee pain	4.040 (0.981, 16.643)	2.473 (0.326, 18.786)	9.990 (1.516, 65.813)
Numbness face	0.000 (0.000, Inf)	0.480 (0.039, 5.954)	0.000 (0.000, Inf)
Numbness hands	1.681 (0.457, 6.179)	1.257 (0.253, 6.243)	2.113 (0.470, 9.493)
Numbness legs	4.200 (1.272, 13.868)	1.025 (0.224, 4.683)	4.304 (1.116, 16.605)
Numbness back	0.166 (0.001, 21.502)	0.393 (0.024, 6.450)	0.065 (0.001, 7.255)
Trembling face	0.000 (0.000, Inf)	6455194000000.000 (0.000, Inf)	0.000 (0.000, Inf)
Trembling hands	2.850 (0.152, 53.518)	0.083 (0.005, 1.464)	0.236 (0.024, 2.331)
Trembling legs	0.000 (0.000, Inf)	21.730 (0.011, 43198.600)	0.000 (0.000, Inf)
Hie general	0.531 (0.169, 1.668)	1.439 (0.407, 5.079)	0.764 (0.206, 2.830)
Hie hands	0.522 (0.141, 1.931)	0.557 (0.159, 1.947)	0.291 (0.077, 1.098)
Hie legs	1.683 (0.630, 4.492)	0.623 (0.204, 1.903)	1.049 (0.352, 3.123)
Hie lower back	1.474 (0.358, 6.075)	0.550 (0.118, 2.562)	0.811 (0.200, 3.291)
Heat face	0.021 (0.004, 0.121)	2.401 (0.724, 7.959)	0.049 (0.008, 0.311)
Heat hands	0.000 (0.000, Inf)	5.293 (0.280, 99.894)	0.000 (0.000, Inf)
Heat legs	0.166 (0.013, 2.050)	12.968 (0.202, 831.143)	2.150 (0.023, 205.044)
Edema face	0.698 (0.141, 3.450)	4.373 (0.392, 48.772)	3.053 (0.271, 34.455)
Edema hands	0.920 (0.126, 6.713)	9.190 (0.226, 373.346)	8.452 (0.215, 332.751)
Edema legs	0.691 (0.246, 1.937)	1.388 (0.429, 4.491)	0.959 (0.300, 3.061)
Headache	0.132 (0.032, 0.545)	3.796 (0.856, 16.839)	0.501 (0.096, 2.605)
Sluggishness	0.176 (0.033, 0.943)	0.700 (0.171, 2.868)	0.123 (0.022, 0.701)
Vertigo	0.747 (0.206, 2.707)	0.938 (0.229, 3.832)	0.701 (0.173, 2.839)
Lightheadedness	0.403 (0.085, 1.906)	0.960 (0.198, 4.648)	0.387 (0.072, 2.066)
Dandruff	94.688 (0.100, 89677.910)	0.008 (0.000, 9.612)	0.826 (0.038, 17.818)
Hair loss	0.286 (0.069, 1.187)	1.316 (0.328, 5.280)	0.376 (0.078, 1.804)
Decreased visual acuity	0.603 (0.194, 1.877)	0.779 (0.228, 2.667)	0.470 (0.138, 1.604)
Eyestrain	0.215 (0.074, 0.627)	1.754 (0.540, 5.702)	0.377 (0.119, 1.189)
Blurred vision	0.616 (0.203, 1.867)	1.718 (0.477, 6.183)	1.058 (0.295, 3.794)
Bleary eyes	0.110 (0.033, 0.367)	2.698 (0.789, 9.228)	0.297 (0.080, 1.102)
Dark circles of the eyes	0.206 (0.049, 0.862)	1.207 (0.344, 4.235)	0.248 (0.053, 1.161)
Sneezing	0.712 (0.162, 3.126)	3.991 (0.505, 31.530)	2.840 (0.361, 22.364)
White nasal discharge (Bi)	0.612 (0.208, 1.799)	0.760 (0.258, 2.236)	0.465 (0.151, 1.433)
Yellow nasal discharge (Bi)	1.732 (0.152, 19.747)	1712.672 (0.000, Inf)	2964.441 (0.000, Inf)
Post nasal drip	0.934 (0.197, 4.425)	0.404 (0.086, 1.897)	0.377 (0.084, 1.691)
Stuffy nose	2.178 (0.511, 9.288)	0.374 (0.078, 1.807)	0.815 (0.207, 3.217)
Nosebleed	0.000 (0.000, Inf)	0.152 (0.001, 29.264)	0.000 (0.000, Inf)
Mouth bitter	0.536 (0.073, 3.919)	0.770 (0.111, 5.315)	0.413 (0.052, 3.257)
Saliva comes out	0.316 (0.007, 15.076)	86710.230 (0.000, Inf)	27731.080 (0.000, Inf)
Throat pain	0.721 (0.165, 3.142)	0.873 (0.181, 4.223)	0.630 (0.130, 3.057)
Throat jams	0.029 (0.002, 0.499)	4.805 (0.583, 39.619)	0.141 (0.006, 3.387)
Thirsty	1.197 (0.377, 3.801)	0.960 (0.255, 3.613)	1.149 (0.323, 4.090)
Dry mouth	0.557 (0.167, 1.859)	0.707 (0.202, 2.478)	0.394 (0.110, 1.414)
Dry lips	0.757 (0.189, 3.030)	0.450 (0.112, 1.811)	0.340 (0.085, 1.365)
Take water often	0.624 (0.198, 1.959)	1.286 (0.357, 4.636)	0.802 (0.223, 2.887)
Tinnitus	1.023 (0.374, 2.796)	1.213 (0.374, 3.933)	1.241 (0.397, 3.876)
Hearing loss	6.628 (1.557, 28.215)	0.634 (0.106, 3.811)	4.205 (0.970, 18.224)
Cough	0.323 (0.032, 3.239)	3.661 (0.227, 59.010)	1.182 (0.063, 22.259)
White sputum (Bi)	4.398 (0.899, 21.512)	0.205 (0.038, 1.087)	0.900 (0.288, 2.808)
Yellow sputum (Bi)	2.647 (0.266, 26.361)	0.344 (0.030, 3.966)	0.912 (0.144, 5.764)
Asthma	0.068 (0.001, 4.411)	687350.400 (0.000, Inf)	46587.550 (0.000, Inf)
Shortness of breath	1.714 (0.370, 7.932)	0.199 (0.042, 0.948)	0.341 (0.086, 1.357)
Palpitation	0.107 (0.021, 0.545)	0.437 (0.116, 1.648)	0.047 (0.009, 0.254)
Chest pain	1.960 (0.181, 21.196)	0.168 (0.016, 1.737)	0.329 (0.047, 2.317)
Burp	0.025 (0.001, 0.949)	1.456 (0.237, 8.942)	0.037 (0.001, 1.542)
Heartburn	0.857 (0.160, 4.580)	1.748 (0.229, 13.308)	1.497 (0.203, 11.033)
Epigastric jamming discomfort	0.889 (0.109, 7.221)	0.285 (0.042, 1.940)	0.253 (0.039, 1.653)
Nausea	0.165 (0.002, 16.148)	0.124 (0.007, 2.196)	0.021 (0.000, 1.437)
Vomiting	0.007 (0.000, Inf)	0.000 (0.000, Inf)	0.000 (0.000, Inf)
Motion sickness	0.498 (0.081, 3.067)	1.386 (0.198, 9.705)	0.690 (0.086, 5.527)
Stomach fullness	1.141 (0.277, 4.691)	0.907 (0.181, 4.530)	1.034 (0.223, 4.806)
Stomach rumbling	0.647 (0.127, 3.288)	0.235 (0.053, 1.044)	0.152 (0.033, 0.705)
Flaturence	1.357 (0.452, 4.078)	0.963 (0.270, 3.434)	1.306 (0.389, 4.385)
Sleepy after eating	0.242 (0.067, 0.881)	2.939 (0.704, 12.275)	0.712 (0.155, 3.274)
Abdominal pain	4.467 (0.333, 59.926)	0.080 (0.006, 1.071)	0.356 (0.055, 2.285)
Abdominal pain fasting (Bi)	4740.774 (0.000, Inf)	0.000 (0.000, Inf)	0.385 (0.061, 2.434)
Abdominal pain after eating (Bi)	524.449 (0.000, Inf)	0.000 (0.000, Inf)	0.188 (0.019, 1.892)
Abdominal pain at upper (Bi)	0.083 (0.000, Inf)	0.000 (0.000, Inf)	0.000 (0.000, Inf)
Abdominal pain at lower (Bi)	3.600 (0.388, 33.412)	0.222 (0.022, 2.238)	0.800 (0.167, 3.822)
Hand stiffness	0.650 (0.194, 2.181)	3.021 (0.604, 15.108)	1.963 (0.388, 9.934)
Lower extremities weakness	5.565 (1.157, 26.752)	0.396 (0.064, 2.432)	2.201 (0.523, 9.265)
Legs fluctuates	7.336 (1.999, 26.919)	0.586 (0.124, 2.765)	4.296 (1.136, 16.249)
Leg spasms	2.550 (0.840, 7.743)	2.151 (0.514, 8.998)	5.486 (1.402, 21.468)
Frost bite	1.469 (0.098, 21.934)	4.616 (0.029, 735.479)	6.778 (0.050, 920.821)
Heavy menstrual flow (Female, Bi)	0.000 (0.000, Inf)	18125.110 (0.000, Inf)	0.045 (0.000, Inf)
Less menstrual flow (Female, Bi)	0.000 (0.000, Inf)	0.840 (0.233, 3.025)	0.000 (0.000, Inf)
Menstruation textile (Female)	0.000 (0.000, Inf)	1.297 (0.176, 9.535)	0.000 (0.000, Inf)
Menstrual pain (Female)	0.000 (0.000, Inf)	4.351 (0.811, 23.337)	0.000 (0.000, Inf)
Irregular menstruation (Female, Bi)	0.000 (0.000, Inf)	1.094 (0.348, 3.442)	0.000 (0.000, Inf)
Delivery (Female, Bi)	0.758 (0.343, 1.675)	0.711 (0.280, 1.804)	0.538 (0.219, 1.324)
Spontaneous abortion (Female, Bi)	0.514 (0.168, 1.573)	1.764 (0.493, 6.306)	0.906 (0.236, 3.484)
Induced abortion (Female, Bi)	1.463 (0.330, 6.482)	0.506 (0.105, 2.430)	0.740 (0.184, 2.978)
Pregnancy toxemia (Female, Bi)	0.849 (0.052, 13.965)	0.711 (0.043, 11.795)	0.604 (0.036, 9.992)
Abnormal bleeding (Female, Bi)	0.000 (0.000, Inf)	1.445 (0.126, 16.595)	0.000 (0.000, Inf)
Traditional pattern diagnosis			
Excess-Deficiency pattern (Five categories)	9.695 (1.270, 74.040)	1313.121 (79.316, 21739.580)	12696.900 (560.943, 287393.200)
Cold pattern (Bi)	0.639 (0.175, 2.340)	1.875 (0.463, 7.596)	1.199 (0.347, 4.141)
Heat pattern (Bi)	0.080 (0.029, 0.226)	3.578 (1.384, 9.247)	0.288 (0.093, 0.888)
Background			
Female sex (Bi)	0.000 (0.000, Inf)	1129.553 (0.000, Inf)	0.118 (0.025, 0.549)
Age	1.142 (1.089, 1.197)	0.985 (0.953, 1.018)	1.125 (1.073, 1.180)
Body mass index	1.092 (0.942, 1.266)	1.924 (1.472, 2.516)	2.102 (1.600, 2.763)

## Abbreviations

BMI: Body mass index
CART: Classification and regression trees
DSS: Decision support system
ICD-10: International statistical classification of diseases and related health problems 10th revision
LOOCV: Leave-one-out cross-validation
SD: Standard deviations
VAS: Visual analogue scale
